# Testing the annual nature of speleothem banding

**DOI:** 10.1038/srep02633

**Published:** 2013-09-16

**Authors:** Chuan-Chou Shen, Ke Lin, Wuhui Duan, Xiuyang Jiang, Judson W. Partin, R. Lawrence Edwards, Hai Cheng, Ming Tan

**Affiliations:** 1 High-Precision Mass Spectrometry and Environment Change Laboratory (HISPEC), Department of Geosciences, National Taiwan University, Taipei 10617, Taiwan ROC; 2 Key Laboratory of Cenozoic Geology and Environment, Institute of Geology and Geophysics, Chinese Academy of Sciences, Beijing 100029, China; 3 Key Laboratory of Humid Subtropical Eco-geographical Processes, College of Geography Science, Fujian Normal University, Fuzhou 350007, China; 4 Institute for Geophysics, Jackson School of Geosciences, University of Texas-Austin, Austin, Texas 78758, USA; 5 Department of Earth Sciences, University of Minnesota, Minneapolis, Minnesota 55455, USA; 6 Institute of Global Environmental Change, Xi'an Jiaotong University, Xi'an 710049, China

## Abstract

Speleothem laminae have been postulated to form annually, and this lamina-chronology is widely applied to high-resolution modern and past climate reconstructions. However, this argument has not been directly supported by high resolution dating methods. Here we present contemporary single-lamina ^230^Th dating techniques with 2σ precision as good as ±0.5 yr on a laminated stalagmite with density couplets from Xianren Cave, China, that covers the last 300 years. We find that the layers do not always deposit annually. Annual bands can be under- or over-counted by several years during different multi-decadal intervals. The irregular formation of missing and false bands in this example indicates that the assumption of annual speleothem laminae in a climate reconstruction should be approached carefully without a robust absolute-dated chronology.

The formation of annual laminae is well-known in many natural materials, such as trees, corals, ice, and lake/marine sediments. Banding in speleothems has also been considered to be annual^1^,^2^, and annual-layer-counted-chronologies^3^,^4^ have been widely applied to reconstructing recent 100s–1000s-year annual-resolved climate histories^5^,^6^,^7^,^8^,^9^,^10^. The technique was also applied to retrieve the exact time spans and transitions of past abrupt climatic/geological events, such as Termination 1 at 14.6–14.5 kyr BP (before AD 1950) (ref 11), the Younger Dryas (YD) from 12.8–11.5 kyr BP (ref 12), and the 8.2-kyr event^13^, to better understand regional hydrological changes and global teleconnections.

In 1960, radiocarbon was used as the first absolute method to evaluate annual bandings in a speleothem from the United States by Broecker and his collogues^1^. Baker *et al*. (ref 2) showed that time spans yielded by counting bands agree those by a thermal-ionization mass-spectrometric (TIMS) ^230^Th dating method with precision of ±62–750 yr for a Holocene stalagmite from the United Kingdom using luminescent laminae. Consistency between ^230^Th chronology with uncertainties as good as ±6–7 yr and the number of calcite-aragonite laminae for a 2000-yr stalagmite in Nepal^5^ and for florescence couplets from a sample from the USA^14^ suggests that the layers may be annual in origin. Betanourt *et al*. argued for annual resolution by comparing stalagmite band thickness and contemporaneous tree-ring records^15^. However, high-precision absolute dating of single laminae in a stalagmite has not been measured to evaluate the assumption that the bands represent annual deposition. In this study, the complicated nature of banding formation is studied by directly comparing the ^230^Th-dates^16^ of 31 single layers in a 300-yr stalagmite collected from Xianren Cave.

## Results

Xianren Cave (24°07′52″N, 104°07′54″E; altitude: 1371 m) has 100% relative humidity in the inner chamber and is located in SE Yunnan Province, South China (Fig. S1). It is located in the Asian monsoon with distinct dry/wet seasons (Fig. S2). More than 75% of the annual precipitation, 1005 (±142) mm (1σ, AD 1971–2000), falls in the summer monsoon season between May and September.

Stalagmite YPXR5, 233 mm in length (Fig. S3), was collected from Xianren Cave in September 2003. This aragonitic stalagmite features (i) clear couplets of compact (high density) and porous (low density) sub-bandings with fast growth rates of 0.5–1.5 mm/yr and (ii) high uranium concentration (8–26 ppm) (Table S1). Scanning electron microscopic (SEM) images show that the sample is ~100% aragonite and contains only 0–0.01% intrusive detritus^17^. With these advantages, this stalagmite is an idea candidate for evaluating the robustness of stalagmite lamina-chronology^3^,^4^ by single-lamina ^230^Th dating techniques with 2σ precision as good as ±0.5 yr (ref 16).

**Figure 1:**
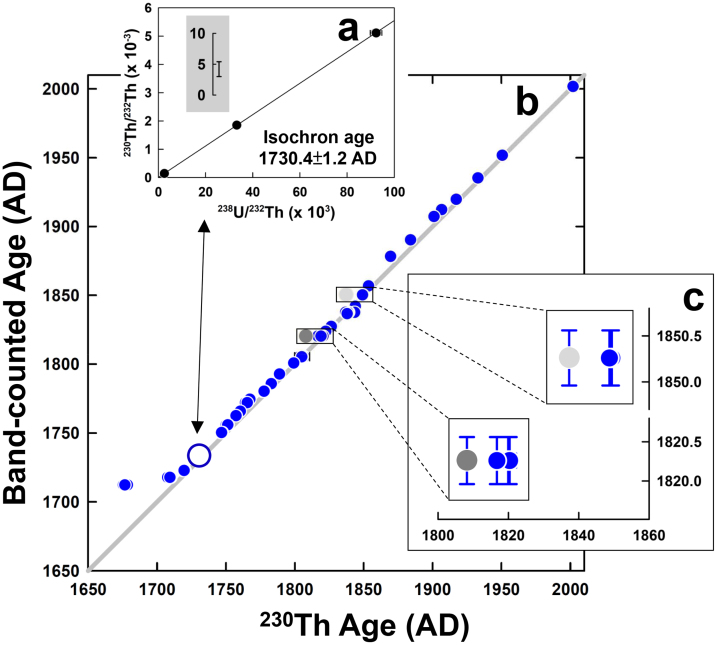
Band-counted and ^230^Th ages. (a) An isochron plot of ^230^Th/^232^Th versus ^238^U/^232^Th (atomic ratios) for four coeval subsamples from a band at 174.0 mm (Fig. S3 and Table S1). An isochron-inferred ^230^Th/^232^Th_0_ atomic ratio (y-intercept with 2σ error) is enlarged in inset. (b) Band-counted age versus ^230^Th age plot for all subsamples of the stalagmite YPXR5 (Fig. S3). ^230^Th dating error bars are smaller than the symbol size for all data. Hollow circle denotes a datum using the isochron age at depth 174.0 ± 0.5 mm. (c) Two discordant ages for subsamples, 10c (gray circle) and 15a (dark gray circle) (Table S1), are 11.9 ± 1.7 and 10.7 ± 2.0 yrs older than those for their coeval subsamples, respectively. Vertical bars represent the correspondent time spans of subsampling widths.

A plot of band-counted age versus ^230^Th age, which is considered as calendar age, is plotted in Figure 1b. The last datum with a banding age of 1712.3 ± 0.5 AD and ^230^Th age of 1676.6 ± 0.8 AD at depth 206.5 ± 0.5 mm does not lie on a 1:1 line due to a clearly visible hiatus at depth 202.0 mm (Fig. S3). The ^230^Th date of the layer at depth 198.0 ± 0.5 mm above the hiatus is 1709.3 ± 1.1 AD. The duration of the growth hiatus is estimated to last ~36 yr. For the segment above the hiatus, data approximately follow the 1:1 line between band-counted age and ^230^Th age (Fig. 1b).

## Discussion

**Figure 2:**
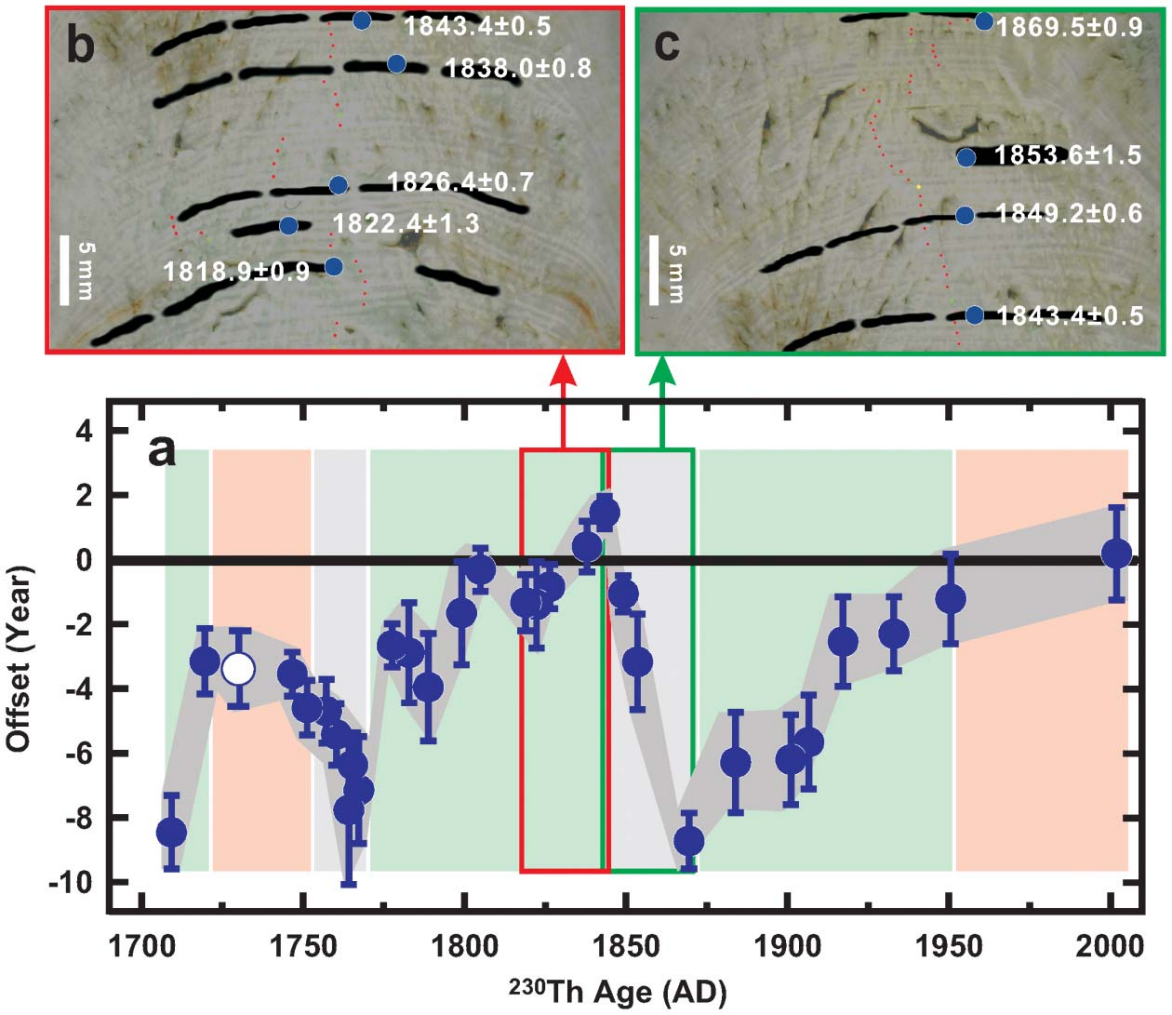
Offset of band-counted age from ^230^Th age. (a) An offset plot of the band-counted age from the ^230^Th age (±2σ error for both variables) from AD 2002–1700 (blue filled circles). Pink, cyan, and gray zones depict the intervals with annual, under-counted and over-counted bands, respectively. The circle with white fill represents the layer with the isochron age from Figure 1a. Examples of stalagmite segments with (b) under-counted (AD 1843–1819) and (c) over-counted (AD 1870–1843) annual bands. Black horizons are subsamples for ^230^Th dating. ^230^Th dates [year (AD) ± 2σ error] are given in white and indicated by a blue circle. Red, green, and yellow dots respectively denote the single, ten, and fifty band counts shown in Fig. S3.

A plot of the offset between the band-counted age and the ^230^Th age for the data above the hiatus at depth 202.0 mm (Figure 2a) clearly shows the discrepancies between the two ages. Offsets of density couplet-inferred ages, ranging from +2 (±1) to −8 (±1) yr (Fig. 2a), cannot be attributed to U-Th chemistry or dating methodology. The chemical procedural ^230^Th blank of 0.0003 ± 0.0003 fmol (ref 16) corresponds to an age error of less than ±0.2 yr, which was corrected during the offline data reduction process^18^. Concordant ^230^Th dates for coeval subsamples at 14 depths (Table S1) further increase our confidence in the U-Th methodology and the estimated ^230^Th/^232^Th_0_ ratio. The only two dates that are exceptions are at depths of 75.5 mm (subsample ID: 10c) and 103.0 mm (subsample ID: 15a), which are different from three other coeval subsamples (Fig. 1c and Table S1). These spurious dates could be biased due to the incorporation of detrital material with a high ^230^Th/^232^Th ratio^19^,^20^ when the stalagmite grew. Therefore, the band-counted age offset in Figure 2a is caused by the complicated formation of these bands where annual bands are sometimes missing, or there are intra-annual (i.e. extra) bands in a year.

For the most recent 52 years, agreement between the number of bands and ^230^Th age (Fig. 2a) indicates that the laminae formed annually at this period, most likely due to the distinct dry/wet seasons from AD 2002–1956 (Fig. S2). Annual banding also occurs in the interval AD 1751–1720. Despite the two periods that agree between the two dating techniques, the offset record is characterized by significant multi-decadal variability. There are two segments with both 8 (±1) under-counted annual bandings during an 80-yr time interval from AD 1950–1870 and a 76-yr interval from AD 1843–1767 (Fig. 2a). On average, the bias of the lamina-chronology is −1 yr per decade for the two sections. In another 11-yr portion of under-counted laminae from AD 1720 to AD 1709, five annual bands are missing.

The number of annual density couplets is over-counted by 10 (±1) during a 27-yr interval from AD 1870–1843 and by 4 (±1) during a 16-yr window from AD 1767–1751. The respective positive biases of +4 and +1 yr per decade are attributed to the formation of intra-annual bands.

**Figure 3:**
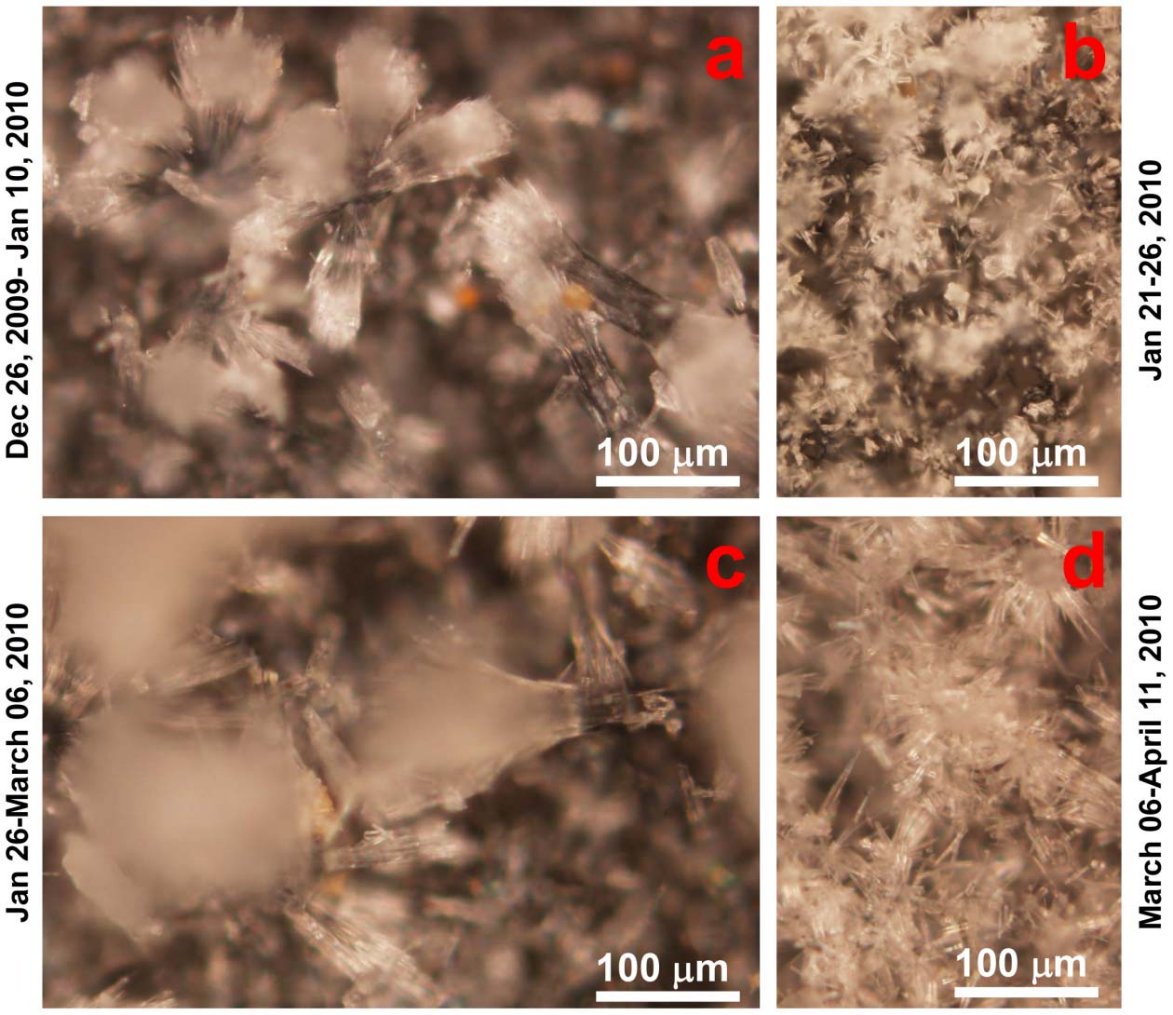
Micrographs of modern aragonite precipitated on glass slides placed at site X13 in Xianren Cave (Fig. 3 of ref 17). Material deposited during the time periods of (a) December 26, 2009-Janunary 10, 2010, (b) January 21–26, 2010, (c) January 26-March 06, 2010, and (d) March 06-April 11, 2010 highlight the different crystal structures of the intra-annual bands. Over the five-month interval, two high density sub-bands composed of columnar structure aragonite [panels (a) and (c)] and two low density sub-bands composed of acicular crystal aragonite [panels (b) and (d)] form the intra-annual banding that would be misidentified as two annual couplets in a lamina-counted chronology.

The missing and intra-annual bands that are identified using high-precision ^230^Th dates are supported by observations made in our *in situ* monitoring program^17^. Carbonate deposition rates were monitored at five sites in the cave for two hydrological years from 2008 September to 2010 July (Fig. S4). Surprisingly, diverse deposition features are displayed. At site X8, carbonate continuously deposited from November 2008 to March 2010. Carbonate formation at sites X6, X7 and X11 is characterized by a missing couplet in the 2nd year. Two annual deposition intervals of November 2008-May 2009 and October 2009-March 2010 are found at site X13. However, during the 2nd deposition interval, multiple, alternating formations of elongated columnar aragonite, the representative crystal for high density sub-bandings, and fine acicular aragonite, the typical structure for low density sub-bandings, from December 2009-April 2010 provides support that intra-annual banding does occur (Fig. 3).

While it is not clear what forces missing and false annual banding, it is likely that different saturation states in the aqueous phase, associated with changes in geochemical and hydrological conditions and CO_2_ degassing are responsible^17^,^21^,^22^. A lack of noticeable hiatuses and indistinguishable micro-crystal structures at all under- and over-counted segments shown in Figure 2b and 2c impede the use of counting stalagmite laminae to construct a faithful chronology.

The example given here shows that speleothem laminae may form annually. It is consistent with the reported annual formation of calcite-aragonite couplets^27^, luminescence bands^2^,^28^, and trace element cycles^29^ in speleothems from locations that are dominated by the seasonal cycle. However, our results also clearly show that complicated deposition of missing and false annual layers can occur in Xianren Cave, a site with a strong seasonal dry/wet cycle, which leads to deviations in the lamina-based age model from the absolute age. While annual band counting and multiproxy studies, such as mineralogy^27^ and/or geochemistry^1^,^28^,^29^, are viable alternative approaches of establishing a speleothem chronology, they may be susceptible to missing or false bands. Therefore, each sample should be carefully evaluated on a case-by-case basis with an independent chronology.

Our findings have important implications for speleothem studies as even a bias of just a few years in a time series^23^ may adversely impact (i) estimates of the timing and duration of abrupt events, such as the YD^12^ and 8.2-kyr event^13^, (ii) studies of annual-to-interannual dynamics of climate systems^7^,^8^,^9^, and (iii) splicing and/or comparing subannual-to-annual resolved records with other proxy records. Careful evaluation^24^ of annual bands should be applied and high-resolution absolute dating^16^ is required to establish robust speleothem age models that reduce the uncertainty in paleoclimatic and paleoenvironmental applications.

## Methods

Sixty eight subsamples, 20–100 mg, were collected from 31 layers of the stalagmite for U-Th chemistry^25^ and isotopic measurements on a multi-collector inductively coupled plasma mass spectrometer (MC-ICP-MS), Thermo Fisher NEPTUNE, in the High-precision Mass Spectrometry and Environment Change Laboratory (HISPEC), Department of Geosciences, National Taiwan University^16^ (Table S1). Among them, 2–4 coeval subsamples from 15 bands were ^230^Th-dated (Fig. S3). Uncertainties in the U-Th isotopic data were calculated at the 2σ level and include corrections for blanks, multiplier dark noise, abundance sensitivity, and contents of nuclides in the spike solution. Except for one subsample, 28b (Table S1), thorium contents are as low as 10s–100s ppt. The initial ^230^Th (^230^Th_0_) levels for most subsamples with these low thorium levels correspond to a bias of only 0.1–0.7 yr.

Ages are corrected for ^230^Th_0_ using an initial ^230^Th/^232^Th (^230^Th/^232^Th_0_) atomic ratio of 4.2 (±1.2) × 10^−6^ (Fig. 1a), obtained with 3-D (^232^Th/^238^U-^230^Th/^238^U-^234^U/^238^U) isochron techniques using *Isoplot* 3.00 by K. R. Ludwig of the Berkeley Geochronology Center^26^ on a layer at depth 174.0 mm (Fig. S3 and Table S1) and an arbitrary variability of 50%. Using this estimated ^230^Th/^232^Th_0_ atomic ratio, the ^230^Th date for the topmost subsample at depth of 0.8 mm is AD 2001.9 ± 1.4, matching the band-counted date of AD 2001.8 ± 0.5 (Table S1). This agreement between the two dating techniques increases our certainty in the calculated ^230^Th/^232^Th_0_ value. Precision ranges from ±1.0 to ±2.0 yr for corrected ^230^Th dates of most subsamples (37/38) and ±0.5 to ±1.1 yr for the weight-averaged dates of 14 layers (Table S1).

## Author Contributions

C.C.S. directed the project; C.C.S. and M.T. designed the experiment. M.T. and W.D. collected the stalagmite. K.L., X.J. and W.D. performed stalagmite banding analysis. C.C.S. and K.L. were responsible for ^230^Th dating. W.D. conducted the *in situ* multi-year field experiment and crystal microstructure analysis. C.C.S., K.L. and X.J. prepared the draft and all authors (C.C.S., K.L., M.T., W.D., X.J., J.W.P., R.L.E. and H.C.) contributed towards preparing the manuscript.

## Supplementary Material

Supplementary Information Supp info
