# Supplementary material for: Testing the annual nature of speleothem banding
Source: Sci Rep. 2013 Sep 16;3:2633. doi: 10.1038/srep02633 (PMC3773624; doi:10.1038/srep02633)
Supplement: Supplementary Information — Supp info [file srep02633-s1.pdf]

SUPPLEMENTARY INFORMATION

Testing the annual nature of speleothem banding

by Shen *et al*., 2013

Supplementary Figures

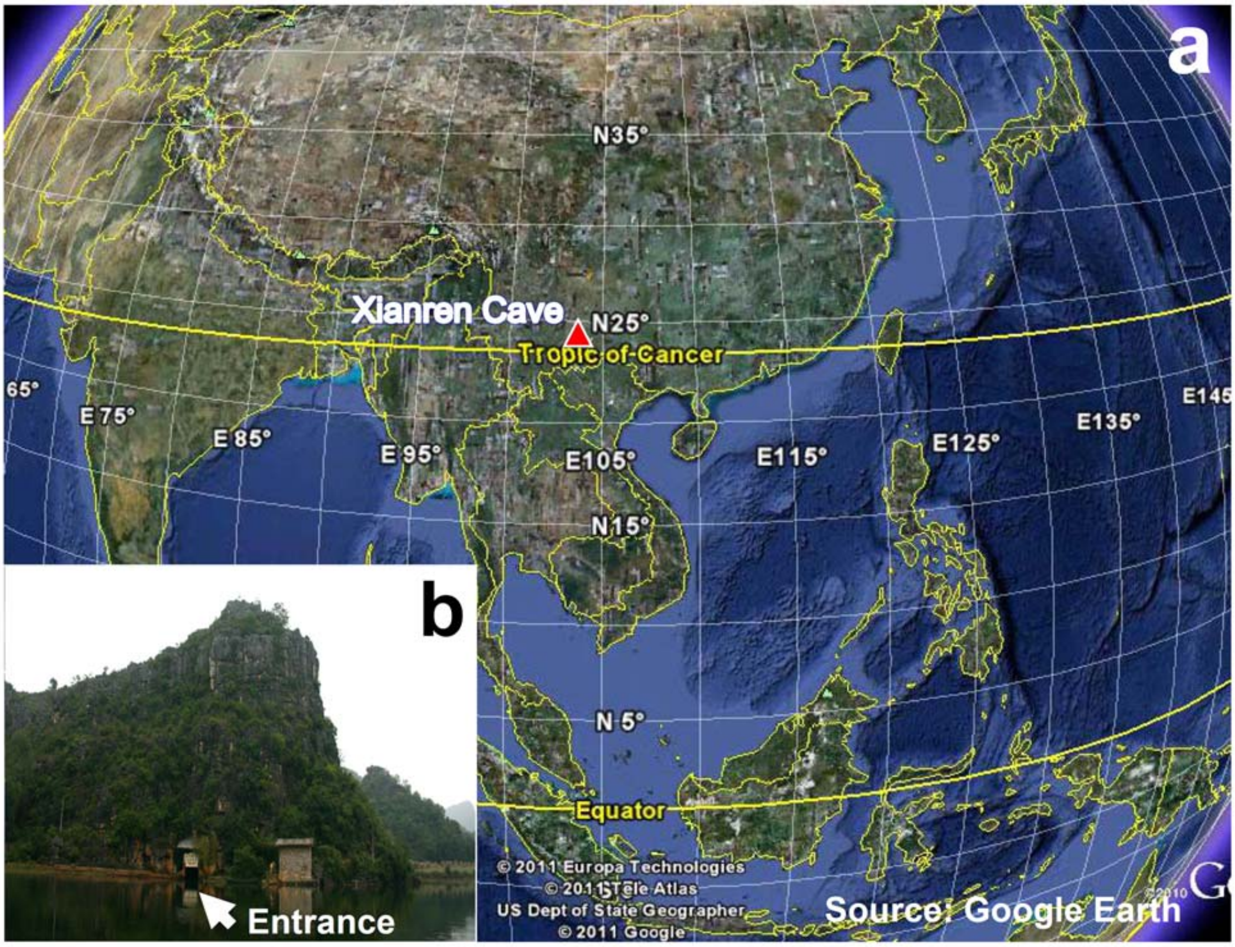

Figure S1. (a) Map with Xianren Cave (triangle), located in South China. [Map source from Google Earth (2011 Europa Technologies, Tele Atlas, US Dept of State Geographer, and Google)] **(b)** A photograph of the middle Triassic host rock, 140 m in height, of Xianren Cave. The arrow denotes the cave entrance. The exact stalagmite YPXR5 sampling location in the cave is available in Figure 3 of ref 17.

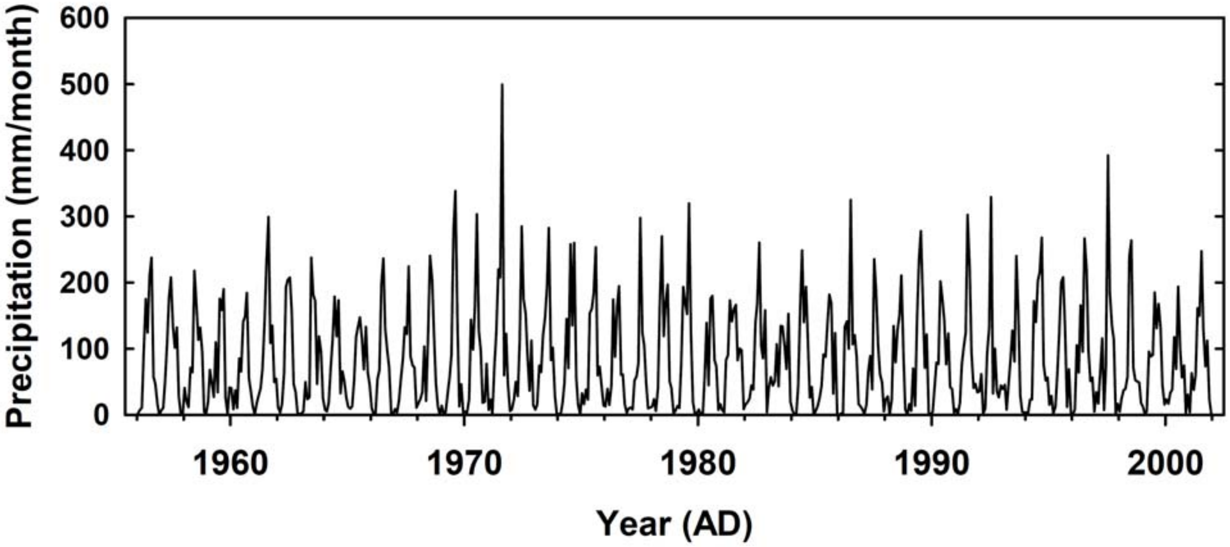

Figure S2. Local monthly precipitation record from AD 1956 to 2002. [Data from a local metrological station, Wenshan (24°07'43''N, 104°08'56''E), 1.7 km SE from Xianren Cave)].

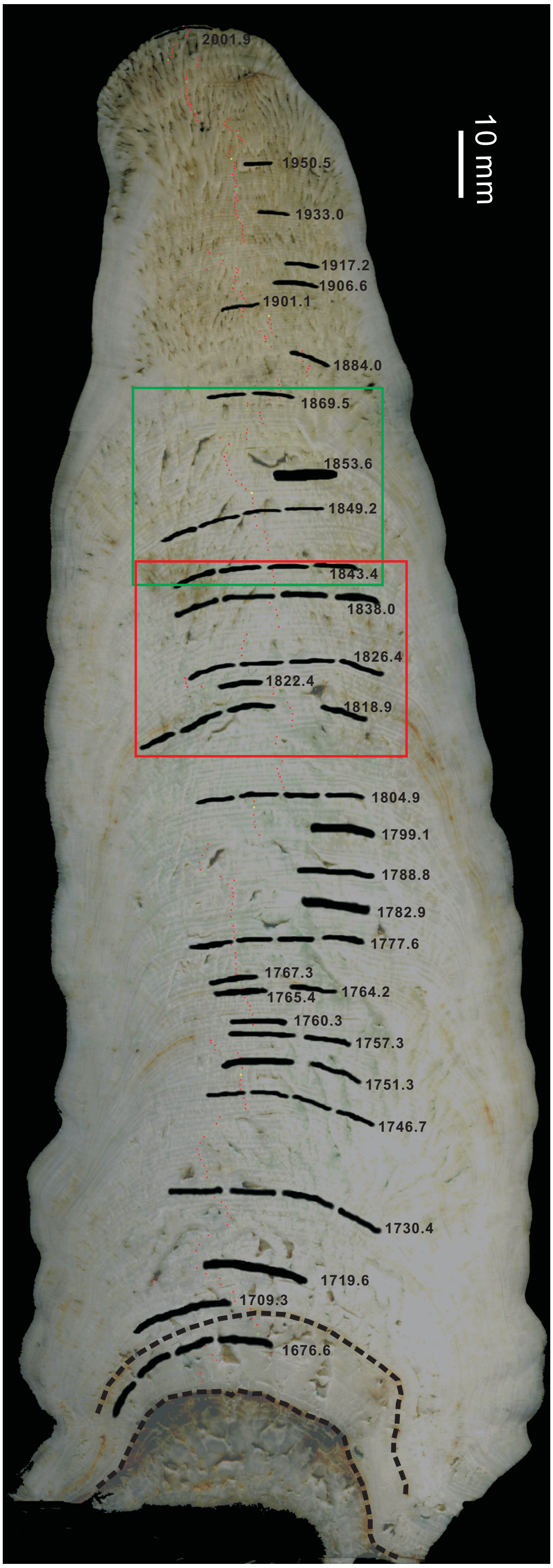

Figure S3. A photograph of the 233.0 mm-long stalagmite, YPXR5, with two distinguishable hiatuses (dashed curves) at depths of 202.0 and 215.0 mm. For ^{230}Th dating[S1], sixty eight single-lamina subsamples, 20-100 mg in weight and 0.6-2.0 mm in width covering ±0.4-1.0 banding, were taken from 31 layers [(black horizons with determined ^{230}Th dates (AD; refer to Table S1 for 2σ error)]. For the 29th (AD 1719.6) and 30th (AD 1709.3) layers, 3 subsamples were split from the drilled powder of single horizon. Isochron techniques were applied to four coeval subsamples, 28a-28d, at depth 174.0 mm (AD 1730.4). Red, green, and yellow dots respectively denote single, ten, and fifty banding counts. The enlarged photographs of two framed areas with red and green lines are given in Figure 2.

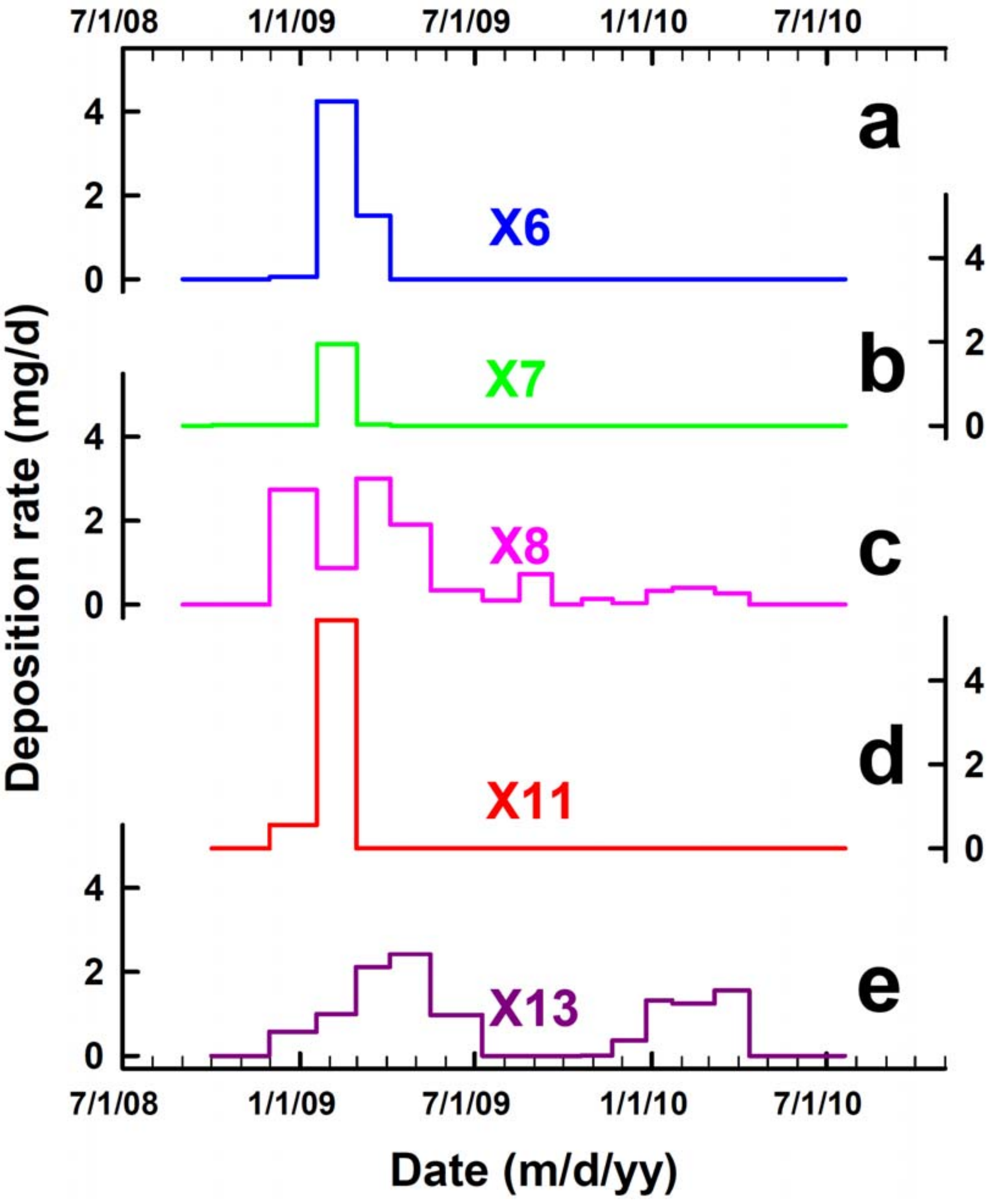

Figure S4. Carbonate deposition rates at five sites, **(a)** X6, **(b)** X7, **(c)** X8, **(d)** X11, and **(e)** X13 (Fig. 3 of ref 17), in Xianren Cave during a 2-year *in situ* monitoring program from September 2008 to July 2010.

Supplementary Tables

Table S1. U-Th isotopic compositions and ^{230}Th ages for subsamples of stalagmite, YPXR5, on MC-ICP-MS at the HISPEC, NTU

**Subsample ID**	**Subsample depth mm**[a]	**Chemistry date AD**	**Weight g**	**^{238}U ppb**	**^{232}Th ppt**	**δ^{234}U measured**[b]	**[^{230}Th/^{238}U] activity**[c]	**[^{230}Th/^{232}Th] ppm**[d]	**Age uncorrected**	**Age corrected**[c,e]	**$\delta^{234}U_{initial}$ corrected**[f]	**^{230}Th date AD**	**Banding #**	**Banding date AD**
**1**	0.8 ± 0.3	2007.9	0.0342	8233 ± 7	441 ± 4	291.7 ± 1.4	0.00008 ± 0.00002	26 ± 5	7.1 ± 1.3	5.9 ± 1.4	291.8 ± 1.4	2,001.9 ± 1.4	2.0 ± 0.5	2,001.8 ± 0.5
**2**	20.0 ± 0.5	2009.9	0.0414	9709 ± 16	106 ± 17	291.2 ± 2.7	0.00070 ± 0.00002	1060 ± 169	59.5 ± 1.4	59.3 ± 1.4	291.3 ± 2.7	1,950.5 ± 1.4	52.0 ± 1.0	1,951.8 ± 1.0
**3**	26.5 ± 0.5	2009.9	0.0435	11699 ± 18	60 ± 16	292.0 ± 2.7	0.00091 ± 0.00001	2944 ± 789	77.0 ± 1.1	76.9 ± 1.1	292.1 ± 2.7	1,933.0 ± 1.1	68.5 ± 0.3	1,935.3 ± 0.3
**4**	37.0 ± 0.5	2009.1	0.0424	8524 ± 12	63 ± 16	292.2 ± 2.4	0.00109 ± 0.00002	2428 ± 633	92.1 ± 1.4	91.9 ± 1.4	292.3 ± 2.4	1,917.2 ± 1.4	84.0 ± 1.0	1,919.8 ± 1.0
**5**	40.0 ± 0.5	2008.0	0.0399	10376 ± 17	97 ± 3	289.5 ± 2.4	0.00120 ± 0.00002	2121 ± 71	101.6 ± 1.5	101.4 ± 1.5	289.6 ± 2.4	1,906.6 ± 1.5	91.5 ± 1.0	1,912.3 ± 1.0
**6**	43.0 ± 0.5	2007.9	0.0429	8396 ± 8	88 ± 3	292.4 ± 1.6	0.00127 ± 0.00002	1997 ± 68	107.1 ± 1.4	106.9 ± 1.4	292.5 ± 1.6	1,901.1 ± 1.4	96.5 ± 0.5	1,907.3 ± 0.5
**7**	47.5 ± 0.5	2009.9	0.0459	9282 ± 14	90 ± 15	290.6 ± 2.5	0.00149 ± 0.00002	2539 ± 429	126.1 ± 1.6	125.9 ± 1.6	290.7 ± 2.5	1,884.0 ± 1.6	113.5 ± 1.0	1,890.3 ± 1.0
**8a**	58.5 ± 0.5	2009.1	0.0546	10718 ± 17	64 ± 13	292.5 ± 2.7	0.00165 ± 0.00001	4553 ± 908	139.2 ± 1.1	139.1 ± 1.1	292.6 ± 2.7	1,870.0 ± 1.1	125.5 ± 0.5	1,878.3 ± 0.5
**8b**		2009.1	0.0354	12947 ± 19	198 ± 20	289.7 ± 2.3	0.00166 ± 0.00002	1788 ± 178	140.7 ± 1.3	140.3 ± 1.4	289.8 ± 2.3	1,868.8 ± 1.4	125.5 ± 0.5	1,878.3 ± 0.5
											**wt-averaged date**	**1,869.5 ± 0.9**	125.5 ± 0.5	1,878.3 ± 0.5
**9**	67.0 ± 1.0	2009.9	0.0468	11799 ± 20	202 ± 15	293.5 ± 2.7	0.00185 ± 0.00002	1792 ± 133	156.6 ± 1.5	156.3 ± 1.5	293.6 ± 2.7	1,853.6 ± 1.5	147.0 ± 1.0	1,856.8 ± 1.0
**10a**	75.5 ± 0.4	2008.3	0.0527	12040 ± 13	38 ± 2	293.2 ± 1.7	0.00188 ± 0.00001	9954 ± 586	159.1 ± 0.9	159.0 ± 0.9	293.3 ± 1.7	1,849.3 ± 0.9	153.5 ± 0.3	1,850.3 ± 0.3
**10b**		2008.3	0.0584	12959 ± 10	15 ± 2	294.2 ± 1.5	0.00188 ± 0.00001	27112 ± 3632	158.9 ± 0.9	158.9 ± 0.9	294.3 ± 1.5	1,849.4 ± 0.9	153.5 ± 0.3	1,850.3 ± 0.3
**10c**		2008.3	0.0447	13843 ± 12	45 ± 3	293.9 ± 1.5	0.00203 ± 0.00002	10358 ± 610	171.1 ± 1.6	171.0 ± 1.6	294.1 ± 1.5	***1837.3*** [g] *± 1.6*	153.5 ± 0.3	1,850.3 ± 0.3
**10d**		2008.3	0.0380	11665 ± 12	40 ± 3	293.8 ± 1.8	0.00189 ± 0.00001	9061 ± 692	159.7 ± 1.2	159.6 ± 1.2	294.0 ± 1.8	1,848.7 ± 1.2	153.5 ± 0.3	1,850.3 ± 0.3
											**wt-averaged date**	**1,849.2 ± 0.6**	153.5 ± 0.3	1,850.3 ± 0.3
**11a**	82.5 ± 0.5	2008.2	0.0294	15884 ± 10	156 ± 4	293.1 ± 1.2	0.00196 ± 0.00001	3289 ± 87	165.6 ± 1.2	165.4 ± 1.2	293.2 ± 1.2	1,842.8 ± 1.2	161.8 ± 0.3	1,842.0 ± 0.3
**11b**		2008.2	0.0226	20431 ± 16	64 ± 5	295.0 ± 1.2	0.00195 ± 0.00001	10334 ± 837	164.7 ± 1.0	164.7 ± 1.0	295.2 ± 1.2	1,843.5 ± 1.0	161.8 ± 0.3	1,842.0 ± 0.3
**11c**		2008.2	0.0321	19666 ± 16	115 ± 4	294.6 ± 1.4	0.00195 ± 0.00001	5498 ± 175	164.9 ± 0.9	164.7 ± 0.9	294.7 ± 1.4	1,843.4 ± 0.9	161.8 ± 0.3	1,842.0 ± 0.3
**11d**		2008.2	0.0270	13411 ± 13	86 ± 4	293.4 ± 1.7	0.00195 ± 0.00001	4991 ± 251	164.4 ± 1.2	164.3 ± 1.2	293.6 ± 1.7	1,843.9 ± 1.2	161.8 ± 0.3	1,842.0 ± 0.3
											**wt-averaged date**	**1,843.4 ± 0.5**	161.8 ± 0.3	1,842.0 ± 0.3
**12a**	86.0 ± 0.5	2009.1	0.0442	10278 ± 14	79 ± 16	293.4 ± 2.6	0.00201 ± 0.00002	4351 ± 873	170.2 ± 1.5	170.1 ± 1.5	293.5 ± 2.6	1,839.1 ± 1.5	166.2 ± 0.5	1,837.6 ± 0.5
**12b**		2009.1	0.0456	12209 ± 18	135 ± 15	291.4 ± 2.6	0.00203 ± 0.00002	3034 ± 344	172.1 ± 1.5	171.9 ± 1.5	291.6 ± 2.6	1,837.3 ± 1.5	166.2 ± 0.5	1,837.6 ± 0.5
**12c**		2009.1	0.0338	11819 ± 15	86 ± 21	292.3 ± 1.9	0.00202 ± 0.00002	4596 ± 1105	170.8 ± 1.6	170.6 ± 1.6	292.5 ± 1.9	1,838.5 ± 1.6	166.2 ± 0.5	1,837.6 ± 0.5
**12d**		2009.1	0.0372	10238 ± 17	52 ± 19	292.7 ± 2.7	0.00204 ± 0.00002	6591 ± 2360	172.3 ± 1.7	172.2 ± 1.7	292.9 ± 2.7	1,836.9 ± 1.7	166.2 ± 0.5	1,837.6 ± 0.5
											**wt-averaged date**	**1,838.0 ± 0.8**	166.2 ± 0.5	1,837.6 ± 0.5
**13a**	96.5 ± 0.5	2009.1	0.0412	17154 ± 32	61 ± 17	292.1 ± 3.0	0.00215 ± 0.00002	10039 ± 2791	182.2 ± 1.3	182.2 ± 1.3	292.3 ± 3.0	1,827.0 ± 1.3	176.5 ± 0.5	1,827.3 ± 0.5
**13b**		2009.1	0.0433	16803 ± 30	144 ± 16	291.7 ± 3.0	0.00217 ± 0.00001	4187 ± 469	183.8 ± 1.3	183.6 ± 1.3	291.8 ± 3.0	1,825.5 ± 1.3	176.5 ± 0.5	1,827.3 ± 0.5
**13c**		2009.1	0.0393	16894 ± 31	361 ± 18	292.8 ± 2.8	0.00217 ± 0.00001	1672 ± 83	183.2 ± 1.2	182.7 ± 1.2	293.0 ± 2.8	1,826.4 ± 1.2	176.5 ± 0.5	1,827.3 ± 0.5
**13d**		2009.1	0.0395	14086 ± 30	75 ± 18	295.6 ± 3.5	0.00216 ± 0.00002	6707 ± 1581	182.0 ± 1.8	181.9 ± 1.8	295.8 ± 3.5	1,827.2 ± 1.8	176.5 ± 0.5	1,827.3 ± 0.5
											**wt-averaged date**	**1,826.4 ± 0.7**	176.5 ± 0.5	1,827.3 ± 0.5
**14**	100.0 ± 1.0	2008.0	0.0445	14440 ± 21	115 ± 3	295.1 ± 2.5	0.00220 ± 0.00002	4568 ± 110	185.8 ± 1.3	185.7 ± 1.3	295.3 ± 2.5	1,822.4 ± 1.3	180.0 ± 0.3	1,823.8 ± 0.3
**15a**	103.0 ± 0.5	2007.9	0.0252	16918 ± 15	187 ± 5	295.2 ± 1.4	0.00237 ± 0.00002	3538 ± 93	199.9 ± 1.8	199.7 ± 1.8	295.4 ± 1.4	***1808.2*** [g] *± 1.8*	183.5 ± 0.3	1,820.3 ± 0.3
**15b**		2007.9	0.0277	12418 ± 12	146 ± 4	295.7 ± 1.7	0.00223 ± 0.00002	3124 ± 93	188.2 ± 1.6	187.9 ± 1.6	295.9 ± 1.7	1,819.9 ± 1.6	183.5 ± 0.3	1,820.3 ± 0.3
**15c**		2007.9	0.0209	17076 ± 20	158 ± 6	298.6 ± 1.9	0.00223 ± 0.00002	3978 ± 145	187.8 ± 1.9	187.6 ± 1.9	298.7 ± 1.9	1,820.3 ± 1.9	183.5 ± 0.3	1,820.3 ± 0.3
**15d**		2007.9	0.0486	9816 ± 14	368 ± 3	293.4 ± 2.3	0.00227 ± 0.00002	1000 ± 11	192.0 ± 1.6	191.2 ± 1.7	293.6 ± 2.3	1,816.7 ± 1.7	183.5 ± 0.3	1,820.3 ± 0.3
											**wt-averaged date**	**1,818.9 ± 0.9**	183.5 ± 0.3	1,820.3 ± 0.3

Table S1 (*Continued*).

Subsample ID	Subsample depth mm[a]	Chemistry date AD	Weight g	^{238}U ppb	^{232}Th ppt	$\delta^{234}U$ measured[b]	[$^{230}Th/^{238}U$] activity[c]	[$^{230}Th/^{232}Th$] ppm[d]	Age uncorrected	Age corrected[c,e]	$\delta^{234}U_{initial}$ corrected[f]	^{230}Th date AD	Banding #	Banding date AD
**16a**	115.5 ± 0.5	2008.3	0.0402	12405 ± 12	68 ± 3	293.9 ± 1.5	0.00239 ± 0.00001	7145 ± 304	201.8 ± 1.2	201.6 ± 1.2	294.1 ± 1.5	1,806.6 ± 1.2	198.5 ± 0.5	1,805.3 ± 0.5
**16b**		2008.3	0.0389	13137 ± 14	29 ± 3	292.7 ± 1.8	0.00242 ± 0.00001	18145 ± 1869	205.0 ± 1.1	204.9 ± 1.1	292.8 ± 1.8	1,803.4 ± 1.1	198.5 ± 0.5	1,805.3 ± 0.5
**16c**		2008.3	0.0555	12309 ± 10	422 ± 3	293.8 ± 1.5	0.00241 ± 0.00006	1161 ± 32	203.7 ± 5.5	203.0 ± 5.5	293.9 ± 1.5	1,805.3 ± 5.5	198.5 ± 0.5	1,805.3 ± 0.5
**16d**		2008.3	0.0394	13442 ± 14	108 ± 3	293.5 ± 1.8	0.00241 ± 0.00001	4965 ± 139	203.5 ± 1.2	203.3 ± 1.2	293.7 ± 1.8	1,805.0 ± 1.2	198.5 ± 0.5	1,805.3 ± 0.5
											**wt-averaged date**	**1,804.9 ± 0.7**	198.5 ± 0.5	1,805.3 ± 0.5
**17**	118.5 ± 0.6	2009.9	0.0471	12854 ± 18	167 ± 15	292.2 ± 2.5	0.00249 ± 0.00002	3179 ± 283	211.0 ± 1.6	210.7 ± 1.6	292.3 ± 2.5	1,799.1 ± 1.6	203.0 ± 0.5	1,800.8 ± 0.5
**18**	125.0 ± 0.5	2009.9	0.0489	12218 ± 17	47 ± 14	291.7 ± 2.4	0.00261 ± 0.00002	11222 ± 3402	221.1 ± 1.7	221.0 ± 1.7	291.9 ± 2.4	1,788.8 ± 1.7	211.0 ± 0.5	1,792.8 ± 0.5
**19**	129.5 ± 0.6	2009.9	0.0412	19019 ± 31	307 ± 17	294.1 ± 2.6	0.00269 ± 0.00002	2753 ± 153	227.3 ± 1.5	227.0 ± 1.6	294.3 ± 2.6	1,782.9 ± 1.6	218.0 ± 0.3	1,785.8 ± 0.3
**20a**	137.0 ± 0.5	2008.2	0.0268	15143 ± 21	71 ± 4	291.6 ± 2.4	0.00274 ± 0.00002	9605 ± 591	231.8 ± 2.0	231.7 ± 2.0	291.8 ± 2.4	1,776.5 ± 2.0	223.5 ± 0.3	1,780.3 ± 0.3
**20b**		2008.2	0.0613	16423 ± 28	273 ± 2	288.7 ± 2.8	0.00273 ± 0.00001	2709 ± 24	231.4 ± 1.3	231.1 ± 1.3	288.9 ± 2.8	1,777.2 ± 1.3	223.5 ± 0.3	1,780.3 ± 0.3
**20c**		2008.2	0.0381	14142 ± 22	167 ± 3	288.8 ± 2.6	0.00272 ± 0.00002	3803 ± 74	230.6 ± 1.4	230.4 ± 1.4	289.0 ± 2.6	1,777.9 ± 1.4	223.5 ± 0.3	1,780.3 ± 0.3
**20d**		2008.2	0.0479	13343 ± 16	117 ± 2	292.4 ± 1.7	0.00272 ± 0.00001	5122 ± 110	230.3 ± 1.1	230.1 ± 1.1	292.6 ± 1.7	1,778.1 ± 1.1	223.5 ± 0.3	1,780.3 ± 0.3
											**wt-averaged date**	**1,777.6 ± 0.7**	223.5 ± 0.3	1,780.3 ± 0.3
**21**	142.5 ± 0.5	2007.9	0.0434	13448 ± 23	223 ± 3	288.6 ± 2.6	0.00284 ± 0.00002	2823 ± 39	241.0 ± 1.6	240.6 ± 1.7	288.8 ± 2.6	1,767.3 ± 1.7	229.3 ± 0.5	1,774.5 ± 0.5
**22**	144.5 ± 1.0	2009.1	0.0348	13266 ± 25	375 ± 20	293.3 ± 3.3	0.00291 ± 0.00003	1695 ± 92	245.5 ± 2.3	244.9 ± 2.3	293.5 ± 3.3	1,764.2 ± 2.3	231.8 ± 0.5	1,772.0 ± 0.5
**23**	145.0 ± 1.0	2008.3	0.0788	11555 ± 14	400 ± 2	294.1 ± 2.0	0.00288 ± 0.00001	1375 ± 7	243.6 ± 0.9	242.9 ± 1.0	294.3 ± 2.0	1,765.4 ± 1.0	232.0 ± 0.5	1,771.8 ± 0.5
**24**	148.5 ± 0.5	2008.3	0.0922	10930 ± 10	50 ± 1	290.2 ± 1.7	0.00293 ± 0.00001	10496 ± 266	248.1 ± 1.0	248.0 ± 1.0	290.4 ± 1.7	1,760.3 ± 1.0	238.0 ± 0.5	1,765.8 ± 0.5
**25a**	151.0 ± 0.5	2008.3	0.0884	10899 ± 8	97 ± 1	293.2 ± 1.2	0.00297 ± 0.00001	5539 ± 80	251.1 ± 1.1	250.9 ± 1.1	293.4 ± 1.2	1,757.4 ± 1.1	241.8 ± 0.5	1,762.0 ± 0.5
**25b**		2009.1	0.0446	9952 ± 15	373 ± 16	292.1 ± 2.5	0.00299 ± 0.00002	1317 ± 56	252.8 ± 2.0	252.0 ± 2.0	292.3 ± 2.5	1,757.1 ± 2.0	241.8 ± 0.3	1,762.0 ± 0.3
											**wt-averaged date**	**1,757.3 ± 1.0**	241.8 ± 0.4	1,762.0 ± 0.4
**26a**	154.5 ± 0.5	2008.3	0.1033	11405 ± 10	57 ± 1	292.1 ± 1.7	0.00304 ± 0.00001	9965 ± 199	256.8 ± 1.0	256.7 ± 1.0	292.3 ± 1.7	1,751.6 ± 1.0	247.9 ± 1.0	1,755.9 ± 1.0
**26b**		2009.1	0.0399	12646 ± 25	122 ± 17	291.9 ± 2.8	0.00306 ± 0.00002	5234 ± 748	259.1 ± 1.8	258.9 ± 1.8	292.1 ± 2.8	1,750.2 ± 1.8	247.9 ± 0.5	1,755.9 ± 0.5
											**wt-averaged date**	**1,751.3 ± 0.8**	247.9 ± 0.5	1755.9 ± 0.5
**27a**	159.0 ± 0.5	2008.2	0.0496	12592 ± 14	55 ± 2	292.5 ± 1.8	0.00309 ± 0.00001	11771 ± 508	261.7 ± 1.2	261.6 ± 1.2	292.7 ± 1.8	1,746.7 ± 1.2	253.5 ± 0.5	1,750.3 ± 0.5
**27b**		2008.2	0.0435	11729 ± 15	191 ± 3	290.5 ± 2.1	0.00310 ± 0.00002	3139 ± 47	262.4 ± 1.4	262.1 ± 1.4	290.7 ± 2.1	1,746.1 ± 1.4	253.5 ± 0.5	1,750.3 ± 0.5
**27c**		2008.2	0.0597	11808 ± 13	60 ± 2	288.5 ± 2.1	0.00308 ± 0.00001	9924 ± 324	261.0 ± 1.3	260.9 ± 1.3	288.7 ± 2.1	1,747.3 ± 1.3	253.5 ± 0.5	1,750.3 ± 0.5
**27d**		2008.2	0.0535	11164 ± 14	39 ± 2	289.7 ± 2.1	0.00309 ± 0.00002	14416 ± 797	261.7 ± 1.6	261.6 ± 1.6	289.9 ± 2.1	1,746.6 ± 1.6	253.5 ± 0.5	1,750.3 ± 0.5
											**wt-averaged date**	**1,746.7 ± 0.7**	253.5 ± 0.5	1,750.3 ± 0.5
**28a**	174.0 ± 0.5	2008.2	0.0225	14557 ± 14	427 ± 5	289.1 ± 1.8	0.00329 ± 0.00002	1853 ± 26	279.4 ± 1.8				270.0 ± 0.5	1,733.8 ± 0.5
**28b**			0.0185	26425 ± 28	10149 ± 19	289.8 ± 1.7	0.00338 ± 0.00003	145 ± 1	286.2 ± 2.2				270.0 ± 0.5	1,733.8 ± 0.5
**28c**			0.0239	19167 ± 18	203 ± 5	290.4 ± 1.6	0.00329 ± 0.00002	5120 ± 126	278.3 ± 1.4				270.0 ± 0.5	1,733.8 ± 0.5
**28d**			0.0242	17295 ± 19	183 ± 5	291.2 ± 1.7	0.00327 ± 0.00002	5111 ± 138	276.7 ± 1.7				270.0 ± 0.5	1,733.8 ± 0.5
									**isochron age**	277.8 ± 1.2	290.6 ± 1.0	1,730.4 ± 1.2	270.0 ± 0.5	1,733.8 ± 0.5
**29a**	184.5 ± 0.5	2007.9	0.0268	20160 ± 22	820 ± 4	291.7 ± 1.6	0.00341 ± 0.00002	1383 ± 11	288.4 ± 1.8	287.5 ± 1.8	292.0 ± 1.6	1,720.4 ± 1.8	281.0 ± 0.5	1,722.8 ± 0.5
**29b**		2007.9	0.0209	22581 ± 31	699 ± 6	287.4 ± 2.3	0.00340 ± 0.00002	1813 ± 18	288.8 ± 1.8	288.2 ± 1.8	287.7 ± 2.3	1,719.7 ± 1.8	281.0 ± 0.5	1,722.8 ± 0.5
**29c**		2007.9	0.0286	18402 ± 23	445 ± 4	288.6 ± 1.9	0.00341 ± 0.00002	2329 ± 26	289.5 ± 1.6	289.0 ± 1.7	288.9 ± 1.9	1,718.9 ± 1.7	281.0 ± 0.5	1,722.8 ± 0.5
											**wt-averaged date**	**1,719.6 ± 1.0**	281.0 ± 0.5	1,722.8 ± 0.5

Table S1 (*Continued*).

Subsample ID	Subsample depth mm[a]	Chemistry date AD	Weight g	^{238}U ppb	^{232}Th ppt	$\delta^{234}U$ measured[b]	$[^{230}Th/^{238}U]$ activity[c]	$[^{230}Th/^{232}Th]$ ppm[d]	Age uncorrected	Age corrected[c,e]	$\delta^{234}U_{initial}$ corrected[f]	^{230}Th date AD	Banding #	Banding date AD
**30a**	198.0 ± 0.5	2007.9	0.0215	15015 ± 19	214 ± 5	286.1 ± 2.2	0.00353 ± 0.00002	4100 ± 107	300.5 ± 2.1	300.2 ± 2.1	286.4 ± 2.2	1,707.7 ± 2.1	286.0 ± 0.3	1,717.8 ± 0.3
**30b**		2007.9	0.0241	16099 ± 20	90 ± 5	286.3 ± 2.1	0.00351 ± 0.00002	10402 ± 563	298.1 ± 1.8	298.0 ± 1.8	286.6 ± 2.1	1,709.9 ± 1.8	286.0 ± 0.3	1,717.8 ± 0.3
**30c**		2007.9	0.0213	16202 ± 19	92 ± 5	286.6 ± 1.8	0.00351 ± 0.00002	10157 ± 604	298.0 ± 2.1	297.8 ± 2.1	286.9 ± 1.8	1,710.0 ± 2.1	286.0 ± 0.3	1,717.8 ± 0.3
											**wt-averaged date**	**1,709.3 ± 1.1**	286.0 ± 0.3	1,717.8 ± 0.3
**31a**	206.5 ± 0.5	2008.2	0.0450	13131 ± 16	222 ± 3	282.0 ± 2.1	0.00390 ± 0.00002	3808 ± 49	332.3 ± 1.7	332.0 ± 1.7	282.2 ± 2.1	1,676.2 ± 1.7	291.5 ± 0.5	1,712.3 ± 0.5
**31b**		2008.2	0.0393	18897 ± 23	278 ± 3	285.0 ± 2.0	0.00390 ± 0.00002	4385 ± 51	332.3 ± 1.4	332.0 ± 1.4	285.2 ± 2.0	1,676.3 ± 1.4	291.5 ± 0.5	1,712.3 ± 0.5
**31c**		2008.2	0.0368	17103 ± 24	202 ± 3	284.1 ± 2.5	0.00388 ± 0.00002	5425 ± 91	330.0 ± 1.6	329.8 ± 1.6	284.4 ± 2.5	1,678.4 ± 1.6	291.5 ± 0.5	1,712.3 ± 0.5
**31d**		2008.2	0.0402	11398 ± 11	744 ± 3	287.0 ± 1.7	0.00393 ± 0.00002	995 ± 6	334.1 ± 1.5	332.7 ± 1.7	287.3 ± 1.7	1,675.5 ± 1.7	291.5 ± 0.5	1,712.3 ± 0.5
											**wt-averaged date**	**1,676.6 ± 0.8**	291.5 ± 0.5	1,712.3 ± 0.5

Analytical errors are 2σ of the mean.

[a] Depth ±½ subsampling width.

[b] $\delta^{234}U = ([^{234}U/^{238}U]_{activity} - 1) \times 1000$.

[c] $[^{230}Th/^{238}U]_{activity} = 1 - e^{-\lambda_{230}T} + (\delta^{234}U_{measured}/1000)[\lambda_{230}/(\lambda_{230} - \lambda_{234})](1 - e^{-(\lambda_{230} - \lambda_{234})T})$, where T is the age.
Decay constants are 9.1577×10^{-6} yr^{-1} for ^{230}Th, 2.8263×10^{-6} yr^{-1} for ^{234}U (ref S2), and 1.55125×10^{-10} yr^{-1} for ^{238}U (ref S3).

[d] The degree of detrital ^{230}Th contamination is indicated by the $[^{230}Th/^{232}Th]$ atomic ratio instead of the activity ratio.

[e] Age corrections were calculated using an $^{230}Th/^{232}Th$ atomic ratio of 4.2 ppm inferred from an isochron at depth 174.0 mm and 50% variability.

[f] $\delta^{234}U_{initial}$ corrected was calculated based on ^{230}Th age (T), i.e., $\delta^{234}U_{initial} = \delta^{234}U_{measured}\ X\ e^{\lambda_{234}*T}$, and T is corrected age.

[g] Dates of subsamples, 10c and 15a, were not used due to possible incorporation of detrital material (see main text).

References

S1. Shen, C.-C. *et al*. High-precision and high-resolution carbonate ^{230}Th dating by MC-ICP-MS with SEM protocols. *Geochim. Cosmochim. Acta* **99**, 71-86 (2012).

S2. Cheng, H. *et al*. The half-lives of uranium-234 and thorium-230. *Chem. Geo*. **169**, 17-33 (2000).

S3. Jaffey, A. H., Flynn, K. F., Glendenin, L. E., Bentley, W. C. & Essling, A. M. Precision measurement of half-lives and specific activities of ^{235}U and ^{238}U. *Phys. Rev.* C4, 1889–1906 (1971).
